# Observations upon the Connexion between External Scrofula and Pulmonary Consumption

**Published:** 1831-02

**Authors:** Jos. Parrish


### SCROFULA AND CONSUMPTION.

*Observations upon the Connexion between External Scrofula and Pulmonary Consumption.* By Jos. Parrish, M.D.*

In almost every disease we observe, at some period of its progress, an effort of nature to effect a cure. I am a full

* North American Med. and Surg. Journal.

believer in the existence of a principle in the human system (call it the *vis medicatrix naturæ,* or by whatever other name you please,) the office of which is to protect the body against the influence of morbific causes, and to repair the injuries which it has been unable to prevent. The surgeon has opportunities every day of witnessing the operation of this principle in the healing of wounds, the reunion of fractured bones, and in various other processes calculated to throw off offending agents, or to obviate their effects. Nor is the physician, perhaps, less frequently called on to observe its influence, though less obvious to the senses, and not so easily traced amidst the complication of symptoms which engage his attention. We have sometimes occasion to notice its operation in consumption. I have before stated my belief that tuberculous phthisis is a scrofulous affection. In this disease, which has generally bid defiance to art, nature has in some instances attempted a cure, and has succeeded. The metastasis of morbid action in cases of acute disease, is one of the most familiar phenomena which we meet with in practice. Every physician of much experience has seen gout and rheumatism fatally translated from external parts, in which they were safe, to internal and vital organs; and the most alarming symptoms often vanish in consequence of a transfer of the same complaints from the head, heart, or stomach, to the extremities. Chronic diseases, I believe, are subject to the same law; and phthisis has been occasionally averted or cured by the spontaneous occurrence of external scrofula.

In the second volume of the Eclectic Repertory, A.D. 1812, I published some cases illustrative of this fact. One of these it will not be amiss to notice in the present place. A coloured woman, thirty years of age, had laboured under a cough for about a year when I was called to visit her. At this time, in connexion with her cough, she had hectic fever, with frequent chills, and I believed her disease to be phthisis pulmonalis in an incipient state. On my first visit, my attention was directed to several scrofulous tumors which were situated upon the thorax near the axilla, and gave her considerable uneasiness. Instead of endeavouring to disperse these tumors, as the patient herself desired, I concluded rather to promote suppuration, and to give a trial to the efficacy of the external disease in removing the internal. Emollient applications were therefore ordered to the tumefied parts, while palliative remedies were prescribed for the cough. Suppuration took place, and the abscesses were opened. A copious discharge was established; and, happily for the patient, there was an evident decline of the pulmonary complaint. I endeavoured to impress her with the absolute necessity of keeping the abscesses open; and she complied with my directions for this purpose. The consumptive symptoms gradually diminished, and at length entirely disappeared. Long after I had ceased regular attendance, I sought her out, and had the pleasure to find her in perfect health. The discharge from the ulcers was maintained for more than two years, and they were not allowed to heal till all danger from the pulmonary disease had vanished.

In addition to this case, I have been consulted, in the course of my practice, by several patients labouring under disease of the lungs, who have been relieved by the occurrence of external scrofula. I recollect particularly the case of a delicate young man, who was brought to me from New Jersey: he had been affected with cough, and had exhibited other symptoms of pulmonary consumption, which had subsided upon the occurrence of an abscess in his side. Efforts had been made to heal the abscess, but happily they had failed. I had no doubt that the preservation of his life depended upon maintaining the discharge, and gave advice accordingly. The case of a young woman in similar circumstances afterwards came under my notice, to whom I gave similar advice. In both instances, health and life, in all probability, depended on the continuance of the external discharge.

If the opinion which I have advanced is correct, it is of the highest importance to watch, in consumptive cases, any tendency to the production of external scrofula, and to cherish it as a friend rather than to dread and endeavour to repel it as an enemy. It may even be desirable, in some instances, to attempt the establishment of a scrofulous affection of the glands, with a view to the relief or cure of the pulmonary complaint. A very common seat of external scrofula is in the neck; and nature points to this as a suitable position for the application of means calculated to induce the disease. The question now occurs, what are these means? by what measures can we excite scrofulous action in the cervical glands? In an individual strongly predisposed to the complaint, as those affected with tuberculous consumption often are, any irritation applied immediately to the part might have the desired effect. But a case occurred to me many years ago, in which I had an opportunity of trying the efficacy of a specific irritation.

In the summer of 1813, I was called to attend a black child, the son of a servant in the family of a respectable merchant of this city. I found it far advanced in consumption, which soon terminated fatally. In the progress of time the parents, who still remained in the same family, and appeared to be favorite servants, had another child, which was attacked with the same disease, accompanied with the same symptoms as in the former case. Considerable solicitude was felt by the master and mistress of the family, as well as by the parents, and my advice was requested. The child had cough and hectic fever, and appeared to be decidedly in the early stage of consumption. I explained my views as to the nature of the disease, and the mode of treatment I proposed to adopt, and was pleased to find a hearty concurrence on the part of those concerned. The child had never been vaccinated, and I determined to avail myself of the opportunity thus afforded of exciting disease in the glands of the neck. With a sharp lancet I made a number of superficial incisions through the skin, commencing behind the ear, and extending downwards in the course of the glands which are usually the seat of scrofula in this part of the body. I then introduced vaccine virus into the incisions through their whole extent, and directed that the child should be brought to me when the vesicles, which would probably result, should have attained their maturity. When I again saw the patient, I was pleased to perceive very extensive vesicles, accompanied with much tumefaction in the surrounding parts. This was the moment for carrying into full effect the plan I had commenced. Instead of allowing the vesicles to take the regular course, to form scabs and pass away, I scarified them with a lancet, and took care to supply them well with stimulating ointment. My object was to keep them sore as long as possible. Directions to this effect were given to the master and parents, and, happily for the child, they were put into operation. Weeks, and even months, elapsed before we allowed the parts behind the ears to heal. Scrofulous tumors made their appearance in the neck; a gradual diminution of the pulmonary affection was perceptible, and the child was at length relieved of all its dangerous symptoms, which, I am happy to add, have never returned.

I tried the same plan in another family in this city; but the patient was in this instance extremely delicate, and the remedy failed.

The result of the first case, though not of itself sufficient to establish the propriety of the mode of practice recommended, merits, I think, some consideration, when taken in connexion with the course which nature herself sometimes pursues in relieving the system of this terrible disease. My own opinion is decided, that, in persons of consumptive habits, any appearance of external scrofula should be encouraged, and its developement favored by artificial means; and the very worst event to a patient so circumstanced would be, the accomplishment of what is often his own eager desire, the dispersion of the glandular swellings which nature has instituted for his relief. Nor do I confine this recommendation to individuals in whom symptoms of phthisis have already made their appearance. It is sufficient that the habit of body, or the family predisposition, should be such as to offer a strong probability that the lungs may become the seat of the disease, unless it be averted by proper management. It is not always easy to carry the practice into effect. External scrofula is in many instances a painful affection, and if it proceed to suppuration and ulceration, is apt to leave unpleasant scars behind it. As it frequently occurs in the neck, and females especially find it difficult to reconcile themselves to the idea of the lasting deformity in which it terminates, the practitioner is frequently solicited to use repellent remedies; nor is it easy to resist compliance. In some instances he may succeed in effecting a dispersion of the tumors; but exactly in proportion as the external disease is driven away by the use of repellents, the danger of the pulmonary affection is increased. Patients have often applied to me in different stages of consumption, whom upon examination I have found to have been subject, at a former period, to glandular swellings about the neck, the dispersion of which has been the signal for the hitherto lurking disease of the chest to display itself. These glandular swellings I have sometimes seen extending downwards in the course of the absorbents, and have traced them in dissection deep into the cavity of the thorax. In the treatment of such cases, when the subject is strongly predisposed to phthisis, I would urge the necessity of firmly resisting the solicitations of the patient, and carefully avoiding the use of means calculated to repel the external affection. Much rather should the tumors be allowed to run their natural course, or even be assisted in their progress by artificial means; and this remark applies with peculiar force to the cases of young people, particularly young females about the period of the appearance of the catamenia.

The only method of treatment justifiable in cases of external scrofula, complicated with consumption, or with consumptive tendencies, is that addressed to the constitution, which, while it subdues the existing scrofulous action, at the same time places the system in the best condition to resist the attack or progress of the pulmonary complaint. To keep the bowels free from disordered accumulations; to administer internally whatever remedies may be calculated to preserve the several functions in a healthy state, and impart a wholesome vigour to the body generally; to direct a free exposure to the pure and fresh air of the country, and the employment of steady exercise: these are measures which, as they counteract that original vice of constitution in which both the external and the internal disease have their source, are not only unobjectionable, but highly advisable in cases of the character above alluded to. I would repeat, however, that while these measures are in operation, we should be careful rather to cherish than repel the scrofulous tumors by local means; and thus keep open a sluice for the morbid disposition of the system to vent itself, so long as it continues to exist, lest it should become accumulated, and turn in upon the lungs with a fatal increase of its terrible energy. I have no doubt that, by a neglect of this caution, many lives have been cut short which might have been considerably protracted; some which might have been ultimately rescued from the disease.

The fistulous sores which so often make their appearance about the anus in consumptive cases, I am disposed to consider somewhat in the same light with external scrofula. When fistula in ano is unattended by any symptoms of phthisis, or by a decided tendency to the disease, I do not hesitate to attempt the cure by an operation; but I believe there is often a connexion between the two complaints, which, when they exist together, should modify the course ordinarily pursued with regard to the external affection.* I am not prepared to say that a successful operation in a case of fistula attended with consumptive symptoms would certainly aggravate these symptoms; but I am inclined to this opinion, and at any rate feel discouraged from operating under such circumstances by other considerations. It is a conclusion drawn from some experience in this complication of disease, that even after an operation, the fistula is much indisposed to heal: and a long confinement to the bed or chamber, with every variety of dressing that can be devised, will often be insufficient to accomplish the desired object.

In a medical gentleman disposed to phthisis, upon whom I operated for a very small fistula, the sore remained open for nearly a year, during which time the pulmonary disease was developed, to which he ultimately fell a victim.

One of the last cases of this kind which occurred to me, afforded me a striking illustration of the difficulty of effecting

* M. Louis, and some other pathologists, have particularly referred to this fact of the connexion between fistula in ano and pulmonary consumption.—Ed.

a cure of fistula when complicated with consumption. A young gentleman of the legal profession applied to me some years since with a fistula in ano, of which he was very desirous to be relieved. I performed the usual operation, and for some time no unusual or unpleasant circumstance occurred. But in the progress of the cure I observed an indisposition in the wound to heal; cough and hectic fever soon afterwards made their appearance, and eventuated in confirmed consumption, of which the patient ultimately died. The fistula never closed.

At present, when a patient in the commencement of consumption, or consumptively disposed, applies to me with a fistula in the region of the anus, instead of immediately operating, I recommend him to use air and exercise, and all other means calculated to improve and confirm his general health; believing that, as in the case of scrofula, when the general disorder of the constitution is corrected, the external affection and the incipient disease of the lungs will be likely to vanish together.

A young gentleman of this city consulted me under the following circumstances. He was affected with cough, frequent pulse, general debility, and other symptoms of incipient phthisis, which was especially to be feared in his case, as he had lost a parent with the disease. In addition to these symptoms he was suffering with a fistula, which, though small, was very troublesome, and of which he was very desirous to be cured. Both he and his friends were anxious for an operation; but, taught by former experience, I resisted their wishes, and by an explanation of my views upon the subject, induced the patient to relinquish his previous intention. It was in the latter part of summer that he applied to me. I told him that his disease was constitutional, and would probably be very slow in healing, even should an operation be performed; that a source of deeper solicitude was the complaint in his breast; that to confine him now, when the winter was approaching, would be to favor the progress of this complaint, which, before the succeeding spring, might proceed too far to be arrested; that his best plan was to exercise freely, and pursue measures calculated to restore his general health, with the return of which he would probably find his fistula relieved. He followed my advice. Business called him to the south, where he passed the winter, travelling much about the country. In the spring he returned wonderfully improved in general health, and with an improvement also of the local affection. The succeeding winter again carried him to the southern states, through the interior of which he travelled very extensively in the pursuit of mercantile objects, with the effect of completely re-establishing his health, which has not subsequently been materially interrupted.

In concluding these brief observations and notices, I wish simply to repeat my conviction of the great importance, in the treatment of tuberculous phthisis, of bearing in mind the connexion subsisting between the disease of the lungs and external scrofula, and of endeavouring to control the former by a temporary encouragement of the latter, while we are using constitutional means for the ultimate conquest of both.

